# Tumor response to radiotherapy and expression of epidermal growth factor receptor mutation and echinoderm microtubule-associated protein-like 4-anaplastic lymphoma kinase rearrangement in patients with non-small cell lung cancer

**DOI:** 10.1186/s43046-022-00130-7

**Published:** 2022-07-11

**Authors:** Nadeem M. S. Nagi, Yasir A. M. Khair, Khamis H. Bakari, Mohamed N. Nagi, Fabian P. Mghanga

**Affiliations:** 1 Department of Oncology, King Khalid Hospital, King Abdulaziz Road, Najran, 61441 Saudi Arabia; 2 Department of Radiology and Medical Imaging, College of Health Sciences, University of Dodoma, Dodoma, Tanzania; 3 Department of Clinical Medicine, Peramiho Institute of Health and Allied Sciences, Songea, Tanzania

**Keywords:** EGFR mutation, EML4-ALK mutation, Lung cancer, Radiotherapy

## Abstract

**Background:**

One of the main limitations of radiation therapy is the resistance of tumor cells. This study aimed at evaluating the relationship between the expression of epidermal growth factor receptor (EGFR) and echinoderm microtubule-associated protein-like 4-anaplastic lymphoma kinase (EML4-ALK) and tumor radiosensitivity in patients with non-small cell lung cancer.

**Methods:**

Medical case files, pathological results for EGFR and EML4-ALK, and computerized tomography scans of patients with NSCLC treated with thoracic radiation therapy were analyzed.

**Results:**

The sample size was 101 patients with mean age 58.43 ± 9.89 years. Statistically significant differences were observed in the mean reduction of long tumor diameter during the early treatment phase in EGFR-positive versus EGFR-negative patients (*p* value = 0.04) and in short tumor diameter during the late treatment phase in EGFR-positive versus EGFR-negative patients (*p* value = 0.04).

**Conclusion:**

Patients with overexpression of EGFR mutations are more radiosensitive during the early treatment phase, and EML4-ALK mutations were less radiosensitive regardless of phases.

## Background

Lung cancer is the leading cause of cancer-related mortality worldwide [1]. Radiotherapy alone or in combination with other treatment modalities are important in the treatment of non-small cell lung cancer (NSCLC) [1]. However, variable responses to radiotherapy have been observed in patients with NSCLC, with some patients attaining an effective local control and others showing relapses even at higher radiation doses [2]. Several biomarkers have been identified as predictors of tumor responses to radiotherapy.

The epidermal growth factor receptor (EGFR) is often expressed at high levels in various types of cancers of epithelial origin [3], and in certain types of tumors, EGFR is mutated [4, 5]. Mutations in the EGFR gene have been reported as major NSCLC driver mutations [5]. More than 10% of patients with NSCLC in Oceania and approximately 50% in Asia-Pacific region have tumors associated with EGFR mutations [6]. The EGFR gene status has been shown to correlate with radiosensitivity in NSCLC patients. In NSCLC patients with EGFR gene mutations, the response to radiotherapy is higher than in those with other EGFR types signifying that EGFR gene status has a role as a biomarker for radiosensitivity in NSCLC patients [7].

Echinoderm microtubule-associated protein-like 4-anaplastic lymphoma kinase (EML4-ALK) is a tyrosine kinase target that is present in various types of cancers, including anaplastic large-cell lymphoma, NSCLC, and pediatric neuroblastoma. EML4-ALK fusion gene results from an inversion in chromosome 2 that contrasted the 5′ end of the EML4 gene with the 3′ end of the ALK gene [8]. EML4-ALK fusion gene has been detected in about 2–7% of patients with NSCLC and expresses a distinct molecular subtype of lung cancer [9]. Mutation detection and other many specific treatment regimens exist for non-small cell lung cancer with these gene mutations [10].

Molecular alterations in NSCLC have been defined as “driver genes,” and they include EGFR and EML4-ALK. The identification of these molecular alterations related to lung cancer can influence therapeutic decision-making and has led to discovery of targeted therapies with specific inhibitor drugs [9]. The relationship between EML4-ALK and EGFR gene mutation has been previously studied. The EML4-ALK fusion gene, for example, is implicated in resistance to EGFR-tyrosine kinase inhibitors (TKIs) [11, 12]. However, only a small fraction of patients have reported to exhibit the coexistence of the EML4-ALK fusion gene and EGFR gene mutation [13, 14].

In this study, we analyzed the relationship between tumor radiosensitivity and the expression of EGFR and EML4-ALK in patients with non-small cell lung cancer treated with thoracic radiotherapy.

## Methods

This retrospective study was conducted to assess the relationship between tumor radiosensitivity and EGFR and EML4-ALK mutations in patients with non-small cell lung carcinoma who received thoracic radiation therapy. We reviewed data of 101 patients with non-small cell lung cancer who presented at the Cancer Center of the University Hospital between January 2013 and October 2015. The inclusion criteria were all patients with non-small cell lung cancer who received thoracic radiation therapy alone or in combination with other treatment modalities (chemotherapy or targeted therapy) and had both EGFR and EML4-ALK serum levels measured; patients who had four CT readings performed at initial diagnosis, just before therapy (pre-treatment CT), and early tumor response; and patients who had late tumor response. Early tumor response was defined as the response around 4 weeks after initiation of therapy, and late tumor response was defined as the response occurring 4 weeks after completion of therapy. Patients’ medical case files were used to extract the following information: age, sex, pathological diagnosis, TNM staging, site of distant metastasis, the long and short diameters of tumors in each CT scan, performance status of the patient, chemotherapy regimen or targeting therapy added to thoracic radiotherapy, radiotherapy dose, existence of EGFR mutations (for common exon 21 L858R mutation and 19 deletions), and the presence of EML4-ALK in the serum. Other detailed medical notes were also obtained, and archived biopsy material was retrieved if available. However, adequate follow-up patients records could not be retrieved to allow for estimation of the overall survival and progression-free survival.

### Statistical analysis

Statistical analysis was performed using SAS software (version 9.3) and GraphPad Prism software (version 5). The comparisons of pre-CT readings were conducted by two independent sample *t*-test or one-way ANOVA. Comparison of reduction values (pre-CT minus post-CT) was used for covariance analysis and controlled effect of pre-CT. *P* values less than or equal to 0.05 were considered as statistically significant.

## Results

### Demography

The analysis included 101 patients whose age ranged between 23 and 78 years. The mean age of patients was 58.43 ± 9.89 years, and the male to female ratio was 1.46:1.0. Of the evaluated patients, 89 (88.11%) had adenocarcinoma, and 12 (11.89%) had squamous cell carcinoma. Patients who received thoracic radiotherapy alone were 54 patients (53.46%), and those who received thoracic radiotherapy in combination with either chemotherapy or targeted therapy were 47 (46.54%) (Table 1).

**Table 1:** Patients characteristics

Characteristics	Number (%)
**Gender**	
Male	60 (59.4%)
Female	41 (40.6%)
**Pathological diagnosis**	
Adenocarcinoma	89 (88.11%)
Squamous cell carcinoma	12 (11.89%)
**Age**	
Mean ± SD	58.43 ± 9.89
Min~max	23.00~78.00
**RT dose**	
Mean ± SD	58.30 ± 7.73
Min~max	34.00~70.00
**EGFR patients**	
Positive	42 (48.27%)
Negative	45 (51.73%)
**EGFR-positive patients**	
EGFR Exon 21	28 (66.66%)
EGFR Exon 19	14 (33.34%)
**EML4-ALK patients**	
Positive	7 (31.81%)
Negative	15 (68.19%)
**Treatment**	
RT	54 (53.46 %)
RT Plus other treatment modalities	47 (46.54%)

### EGFR and EML4-ALK expression

Eighty-seven patients expressed EGFR mutations; of these, 42 (48.27%) had EGFR-positive mutation, and 45 (51.73%) had EGFR-negative mutation. Fourteen patients (33.34%) had EGFR Exon 19, and 28 patients (66.66%) had EGFR Exon 21. The total number of patients tested for EML4-ALK was 68 patients. Of these, 61 patients were EML4-ALK negative, and 7 (31.81%) patients had positive EML4-ALK mutations. In order to keep the sample size statistically relevant, 15 EML4-ALK-negative patients (68.19%) were randomly selected to be included in our analysis (Table 2).

**Table 2:** Outcome of thoracic radiotherapy based on EGFR expression, EML4-ALK rearrangement, and treatment modalities

Categorical variables			Mean	Mean diff.	95% CI	***p value***
Early response	Reduction in long diameter	EGFR positive (*n* = 42)	0.99	0.41 ± 0.19	0.02–0.79	0.04
		EGFR negative (*n* = 45)	0.58			
	Reduction in short diameter	EGFR positive (*n* = 42)	0.64	0.17 ± 0.21	−0.25–0.59	0.42
		EGFR negative (*n* = 45)	0.47			
Late response	Reduction in long diameter	EGFR positive (*n* = 42)	1.30	0.33 ± 0.27	−0.21–0.87	0.23
		EGFR negative (*n* = 45)	0.97			
	Reduction in short diameter	EGFR positive (*n* = 42)	1.35	0.59 ± 0.28	0.03–1.15	0.04
		EGFR negative (*n* = 45)	0.76			
Early response	Reduction in long diameter	ALK positive (*n* = 7)	0.23	0.21 ± 0.27	−0.78–0.36	0.45
		ALK negative (*n* = 15)	0.44			
	Reduction in short diameter	ALK positive (*n* = 7)	0.16	0.34 ± 0.36	−1.08–0.42	0.37
		ALK negative (*n* = 15)	0.49			
Late response	Reduction in long diameter	ALK positive (*n* = 7)	0.72	0.36 ± 0.42	1.25–0.53	0.40
		ALK negative (*n* = 15)	1.08			
	Reduction in short diameter	ALK positive (*n* = 7)	0.66	0.18 ± 0.53	−1.32–0.95	0.74
		ALK negative (*n* = 15)	0.80			
Early response	Reduction in long diameter	RT (*n* = 54)	0.55	−0.34 ± 0.17	−0.68–0.01	0.05
		RT Plus (*n* = 47)	0.89			
	Reduction in short diameter	RT (*n* = 54)	0.41	−0.19 ± 0.18	−0.16–0.56	0.27
		RT Plus (*n* = 47)	0. 61			
Late response	Reduction in long diameter	RT (*n* = 54)	0.92	0.42 ± 0.26	−0.09–0.94	0.10
		RT Plus (*n* = 47)	1.34			
	Reduction in short diameter	RT (*n* = 54)	0.74	0.27 ± 0.23	−0.73–0.19	0.24
		RT Plus (*n* = 47)	1.02			
Early response	Reduction in long diameter	EGFR positive (*n* = 42)	0.99	0.76 ± 0.42	−0.07–1.60	0.07
		ALK positive (*n* = 7)	0.23			
	Reduction in short diameter	EGFR positive (*n* = 42)	0.64	−0.48 ± 0.41	−1.32–0.35	0.25
		ALK positive (*n* = 7)	0.16			
Late response	Reduction in long diameter	EGFR positive (*n* = 42)	1.30	0.58 ± 0.49	−0.42–1.59	0.25
		ALK positive (*n* = 7)	0.72			
	Reduction in short diameter	EGFR positive (*n* = 42)	1.35	−0.69 ± 0.58	−1.88–0.50	0.25
		ALK positive (*n* = 7)	0.66			
Early response	Reduction in long diameter	EGFR exon 21 (*n* = 28)	1.06	0.21 ± 0.39	−0.58–0.99	0.60
		EGFR exon 19 (*n* = 14)	0.86			
	Reduction in short diameter	EGFR exon 21 (*n* = 28	0.73	0.22 ± 0.42	−0.65–1.01	0.60
		EGFR exon 19 (*n* = 14)	0.51			
Late response	Reduction in long diameter	EGFR exon 21 (*n* = 28)	1.35	0.12 ± 0.45	−0.80–1.05	0.79
		EGFR exon 19 (*n* = 14)	1.23			
	Reduction in short diameter	EGFR exon 21 (*n* = 28)	1.65	0.99 ± 0.50	−0.06–2.05	0.06
		EGFR exon 19 (*n* = 14)	0.65			
Early response	Reduction in long diameter	EGFR exon 21 (*n* = 28)	1.06	0.48 ± 0.21	0.07–0.90	0.02
		EGFR negative (= 45)	0.58			
	Reduction in short diameter	EGFR exon 21 (*n* = 28)	0.73	0.26 ± 0.23	−0.21–0.73	0.27
		EGFR negative (= 45)	0.47			
Late response	Reduction in long diameter	EGFR exon 21 (*n* = 28)	1.35	0.38 ± 0.29	−0.20–0.96	0.20
		EGFR negative (= 45)	0.97			
	Reduction in short diameter	EGFR exon 21 (*n* = 28)	1.65	0.91 ± 0.32	0.27–1.55	0.01
		EGFR negative (= 45)	0.76			
Early response	Reduction in long diameter	EGFR exon 19 (*n* = 14)	0.86	0.28 ± 0.26	−0.25–0.79	0.29
		EGFR negative (= 45)	0.58			
	Reduction in short diameter	EGFR exon 19 (*n* = 14)	0.51	0.04 ± 0.26	−0.48–0.56	0.88
		EGFR negative (= 45)	0.47			
Late response	Reduction in long diameter	EGFR exon 19 (*n* = 14)	1.23	0.26 ± 0.35	−0.45–0.96	0.47
		EGFR negative (= 45)	0.97			
	Reduction in short diameter	EGFR exon 19 (*n* = 14)	0.65	−0.11 ± 0.27	−0.67–0.44	0.68
		EGFR negative (= 45)	0.76			

### Tumor size reduction and expression of EGFR

The outcome of radiotherapy in patients with EGFR positive was compared to that of EGFR-negative patients. We observed a statistically significant difference in the mean reduction of long tumor diameter in the early tumor response (0.99 cm) in EGFR-positive patients compared to 0.58 cm in EGFR-negative patients (*p* value = 0.04). Similarly, a statistically significant difference was also observed in the mean reduction in short tumor diameter (1.35 cm) in EGFR-positive patients compared to 0.76 cm in EGFR-negative patients (*p* value *=* 0.04) during the late tumor response (Table 2*)*.

### Tumor size reduction and expression of EML4-ALK

No significant differences were observed in mean reduction in the long tumor diameters during both early and late tumor responses in EML4-ALK-positive and EML4-ALK-negative patients (*p* value 0.45 vs *p* value 0.40); similarly, no differences were observed when the mean reduction in the short tumor diameter in EML4-ALK-positive patients was compared to that of EML4-ALK-negative patients in both early and late treatment phases (*p* value 0.36 versus *p* value 0.74). When the outcome of radiotherapy effect between EML4-ALK-positive and EML4-ALK-negative patients was evaluated, there were no significant differences in the reduction of the tumor size between the long and short diameters of the tumor in both early (*p* value 0.45 vs *p* value 0.37) and late tumor responses (*p* value 0.40 vs *p* value 0.74) (Table 2).

### Tumor size reduction and treatment modalities

The mean reduction in the long tumor diameter in the early response was 0.55 cm in radiotherapy alone and 0.89 cm in radiotherapy combined with other treatment modalities (chemotherapy or targeted therapy) (*p* value 0.05). On the other hand, there were mean reductions of 0.41 cm and 0.61 cm in the short diameter of the tumors in radiotherapy and combined therapy, respectively (*p* value 0.27). During the late tumor response, mean reductions of 0.92 cm and 1.34 cm were observed in the long diameters of the tumor during radiotherapy and combined therapy, respectively (*p* value 0.10), and 0.74 cm and 1.02 cm mean reductions in short diameters in radiotherapy and combined therapy, respectively (*p* value 0.24) (Table 2).

### Tumor size reduction and expression of EGFR and EML4-ALK

**Fig. 1:**
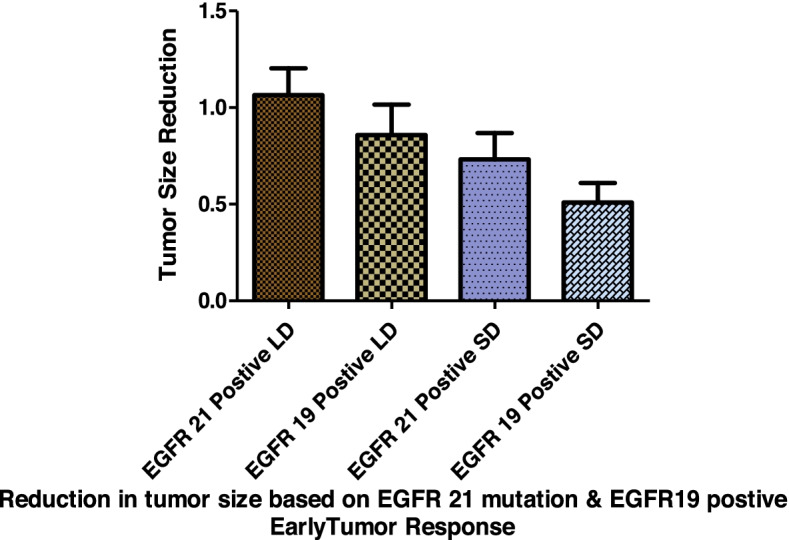
Reduction in tumor size based on EGFR exon 21 and EGFR exon 19 mutations at early tumor response. LD, long diameter of the tumor axis; SD, short diameter of the tumor axis

The outcomes of thoracic radiation therapy between EGFR-positive and EML4-ALK-positive mutation patients during both early and late tumor responses were also compared. During the early tumor response, mean reductions of 0.99 cm and 0.23 cm were observed in long tumor diameters of EGFR-positive patients and EML4-ALK-positive patients (*p* value 0.07), respectively, and reductions of 0.64 cm and 0.16 cm were recorded in short tumor diameters of EGFR-positive patients and EML4-ALK-positive patients (*p* value 0.25), respectively (Fig. 1). During the late tumor response, the recorded mean tumor reductions in the long diameters were 1.30 cm in EGFR and 0.72 cm in the EMLK4-ALK-positive patients (*p* value 0.25) and 1.35 cm and 0.66 cm in the short tumor diameters of EGFR-positive and EML4-ALK-positive patients (*p* value 0.25) (Table 2).

### Tumor size reduction and types of EGFR mutations

EGFR-positive patients were subdivided into two subgroups, EGFR exon 21 (28 patients) and EGFR exon 19 (14 patients), and the treatment outcomes between them were compared. There was, however, no significant difference of the thoracic radiotherapy outcome between the two groups. The result might be affected by the variation in the number of patients which was notably less in the EGFR exon 19 subgroup. The treatment outcome in patients with EGFR exon 19 mutation was compared to that of patients with no EGFR expression, and we found no statistical differences in the treatment outcome between the two groups in both early and late tumor responses (Table 2).

**Fig. 2:**
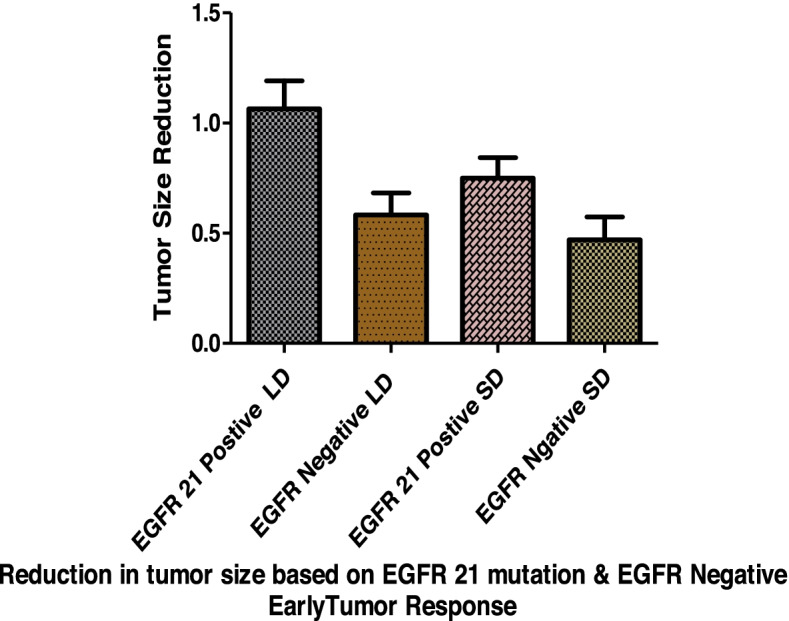
Reduction in tumor size based on EGFR 21 mutation and EGFR negative early tumor response. LD, long diameter of the tumor axis; SD, short diameter of the tumor axis

The outcome of radiotherapy in patients with EGFR exon 21 was compared to that of EGFR-negative patients. We observed a statically significant difference in the mean reduction of long tumor diameter in the early tumor response (1.06 cm) in EGFR exon 21 patients compared to 0.58 cm in EGFR-negative patients (*p* value = 0.02). Similarly, a statistically significant difference was also observed in the mean reduction in short tumor diameter during the late tumor response (1.65 cm) in EGFR exon 21 patients compared to 0.76 cm in EGFR-negative patients (*p* value = 0.01) (Fig. 2 and Table 2).

## Discussion

Thoracic radiotherapy plays a significant role in the treatment of patients with non-small cell lung cancer. In early cancer stages, it can be a suitable alternative option to surgical resection, and in late stages, it has been proved to increase the overall patient survival [11]. Despite being widely used in the treatment of non-small cell lung cancer patients, thoracic radiation therapy is limited by tumor cell resistance and toxicity. In the current study, we evaluated whether the presence or absence of biological markers (EGFR and EML4-ALK) plays a role in affecting radiosensitivity of non-small cell lung cancer cells to radiation therapy.

Endothelial growth factor receptor (EGFR) is among the major driver genes of lung cancer, and its signaling pathway is one of the most broadly studied pathways in human cancers [6]. In this study, we assessed the relationship between the expression and mutations of EGFR and EML4-ALK biomarkers and the possible association with radiosensitivity and the difference in the treatment outcome when using only local thoracic radiation therapy alone or in combination with other treatment modalities.

In this retrospective study, EGFR-positive patients were more sensitive to radiation therapy, with both long and short tumor diameters reduced more significantly than in EGFR-negative patients in both early and late tumor response phases.

Previous studies evaluated the presence of EGFR mutation in irradiated cells. One study investigated this relationship in NSCLC patients with brain metastasis treated with whole brain radiation therapy [15] and concluded that patients with mutant EGFR responded better to brain radiation therapy than those with wild-type EGFR. Although our site of radiation therapy was different (thoracic region versus brain) and our study had a relatively larger number of patients, results in the two studies are similar. Similarly, another study that evaluated the effect of EGFR mutation in the treatment outcome of radiation therapy in patients with locally advanced NSCLC reported favorable outcomes in EGFR mutant patients [16].

There was no difference in the treatment outcome among EML4-ALK-positive and EML4-ALK-negative patients, which might partially be explained by the small number of EML4-ALK-positive patients (7 patients) observed in this study compared to 15 EML4-ALK-negative patients. The smaller number of EML4-ALK-positive patients reported in this study is consistent with those reported in previous studies elsewhere which showed that EML4-ALK is positive in only 2–7% of patients [11, 12, 17]. Similarly, no noticeable differences in the outcomes were observed when treatment outcome in EGFR-positive patients was compared with those with EML4-ALK-positive patients.

Using EGFR-TKI has been shown to have better clinical outcomes in patients with NSCLC [12, 13]. Due to the limited number of patients treated with TKI in this study, it was not possible to assess the effects of adding TKI to thoracic radiation.

We also observed a significant difference in the treatment outcome during early tumor response in patients with EGFR (exon 21 deletion) compared to patients with no EGFR mutation. This was different in patients with EGFR (exon 19 deletion) when their treatment outcome was compared to that of patients with EFGR negative, which may partly be explained by the smaller number of patients with EGFR (exon 19 deletion). Furthermore, patients with mutant EGFR 21 had higher response rate to thoracic radiation therapy. On the other hand, when EGFR 19 and EGFR 21 were compared to each other, no significant difference in the local control outcome was observed.

This present study is limited. First, by absence of some important patients’ information that could not be retrieved from case files; increasing chances of selection bias. With adequate follow-up records, for example, the study could provide information on the estimation of the overall survival and progression-free survival of patients after the complete course of therapy. Secondly, the study was conducted at only one facility, with a relatively small number of assessed patients; therefore, further studies should be conducted at multiple facilities to produce generalizable results.

## Conclusion

Patients with overexpression of EGFR mutations are more radiosensitive during the early treatment phase, and those with EML4-ALK mutations were less radiosensitive regardless of phases. Nevertheless, our findings need confirmation in a future prospective study and necessitate the need for future therapeutic trials to consider types and subtypes of EGFR mutation as important factors to be considered in treatment.

## Data Availability

All data and materials involved in this research are available upon request.
